# Toward Precision Electrochemical Sensing of CFTR Function in Cystic Fibrosis Models

**DOI:** 10.1021/acs.analchem.6c00392

**Published:** 2026-06-04

**Authors:** Antonella Miglione, Giovanna Blaconà, Sima Singh, Stefania Lo Cicero, Andrea Fuso, Giuseppe Cimino, Adriana Eramo, Umberto Malapelle, Marco Lucarelli, Stefano Cinti

**Affiliations:** † Department of Public Health, 9307 University Federico II of Naples, 80138 Naples, Italy; ‡ Department of Experimental Medicine, 9311 Sapienza University of Rome, 00161 Rome, Italy; § Department of Pharmacy, University of Naples Federico II, Via D. Montesano 49, 80131 Naples, Italy; ∥ Department of Oncology and Molecular Medicine, 9289 Istituto Superiore di Sanità, 00161 Rome, Italy; ⊥ CRiN, Center for Research in Neurobiology, Sapienza University of Rome, 00185 Rome, Italy; # Cystic Fibrosis Centre, 18652 AOU Policlinico Umberto 1, 00161 Rome, Italy; ∇ Pasteur Institute Cenci Bolognetti Foundation, Sapienza University of Rome, 00185 Rome, Italy; ○ Bioelectronics Task Force at University of Naples Federico II, Via Cinthia 21, 80126 Naples, Italy; ◆ Sbarro Institute for Cancer Research and Molecular Medicine, Center for Biotechnology, College of Science and Technology, Temple University, Philadelphia, Pennsylvania 19122, United States

## Abstract

The assessment of cystic fibrosis transmembrane conductance regulator (CFTR) activity is essential to both diagnose and evaluate the efficacy of treatments in cystic fibrosis (CF). In particular, the prompt evaluation of genotype-specific responses to CFTR modulators might lead to more precise therapeutic approaches. In this study, a flexible electrochemical sensor modified with silver nanoparticles was developed and applied for the electrochemical quantification of chloride ions in CF epithelial cell models. The sensing platform exhibited a linear analytical response toward chloride in both aqueous solutions and chloride-free buffer, with limits of detection in the submillimolar range, enabling the discrimination of chloride concentrations under various conditions. Chloride ions were detected in apical surface liquids obtained from epithelial cultures carrying different CFTR pathogenic variants: the effect of the elexacaftor/tezacaftor/ivacaftor (ETI) treatment, as a CFTR modulator, was differentiated from untreated samples. Chloride concentrations were determined by differential pulse voltammetry using the standard addition method, and results were further normalized to express chloride secretion considering the final apical fluid volume. ETI treatment induced genotype-dependent increases in chloride secretion, with the largest functional recovery observed in responsive genotypes such as F508del/F508del and L1077P/L1077P. A strong correlation was found between chloride levels and normalized secretion rates, confirming how direct electrochemical measurements reliably reflect CFTR functional changes. Overall, this work demonstrates that portable sensors provide a rapid, cost-effective, and scalable approach for functional CFTR evaluation, supporting their potential application in the personalized assessment of CFTR modulator efficacy, namely theratyping.

## Graphical abstract

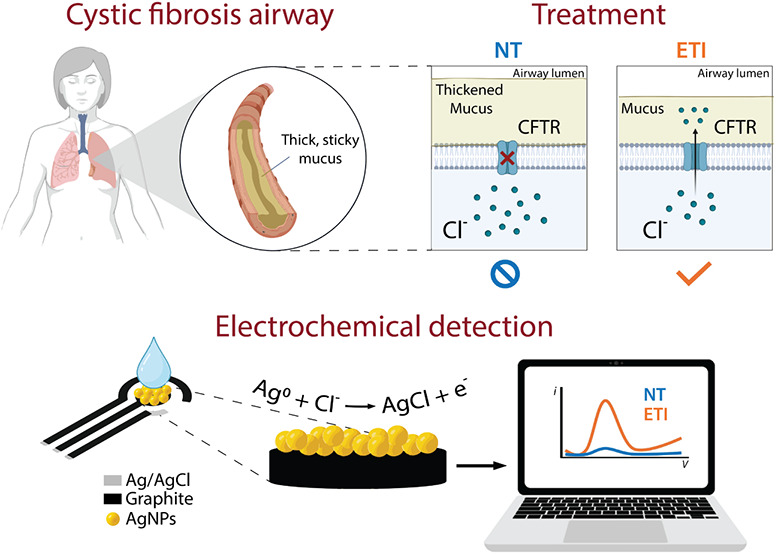

## Introduction

Cystic fibrosis (CF) is a life-limiting genetic disorder caused by pathogenic variants in the cystic fibrosis transmembrane conductance regulator (CFTR) gene, which encodes a chloride (Cl^–^) and bicarbonate channel expressed on the apical membrane of epithelial cells.^1–3^ CFTR plays a pivotal role in controlling chloride and bicarbonate secretion, epithelial hydration, and mucociliary clearance in several organs, including the airways, intestine, and pancreas. Impaired CFTR function results in defective chloride secretion and dysregulated epithelial ion transport, ultimately leading to dehydrated epithelial surface liquid and viscous mucus accumulation.^3^ Chloride (Cl^–^) is the major anion secreted by epithelial cells and is a key determinant of epithelial surface liquid composition and volume.^4^ In CF epithelia, reduced CFTR-mediated chloride transport is accompanied by increased sodium absorption through the epithelial sodium channel (ENaC), further exacerbating fluid reabsorption from the epithelial surface. This altered ion and fluid homeostasis underlies many of the hallmark features of CF, including impaired mucociliary clearance, chronic bacterial colonization, and progressive tissue damage.^5,6^

The worldwide prevalence of CF has been documented at 188,336 cases spread across 96 countries with 111,767 confirmed diagnoses recorded so far.^7,8^ Notably, the CF population is progressively aging due to improved clinical management and the advent of CFTR modulator therapies. In Europe, the adult CF population increased by approximately 60% over the past decade, reaching more than 21,000 people with CF (pwCF) in 2021.^9,10^

At the molecular level, CF arises from pathogenic variants in the CFTR gene located on chromosome 7q31.2.^2^ Among the more than 2,000 CFTR variants identified, the F508del is the most prevalent and results in protein misfolding, impaired trafficking to the plasma membrane, and premature degradation.^11,12^ The consequent loss of CFTR function compromises chloride secretion and its inhibitory control over ENaC, leading to excessive sodium absorption, depletion of epithelial surface liquid, and formation of dense mucus.^5^ These pathophysiological alterations promote chronic bacterial infection, most notably by *Pseudomonas aeruginosa*, and drive the progressive pulmonary, pancreatic, and multisystem manifestations characteristic of CF.^13^ Other pathogenic variants, such as L1077P, W1282X, S589I, and G85E, are associated with variable residual CFTR activity and heterogeneous responses to pharmacological treatments.^12,14–16^

Given the central role of CFTR in chloride transport, the assessment of chloride flux in biological fluids derived from epithelial surfaces has therefore become a key functional readout of CFTR activity.^17^ In clinical practice, sweat chloride concentration is widely used for CF diagnosis, reflecting the inability of CFTR-defective sweat ducts to reabsorb chloride.^18^ Sweat chloride values above 60 mmol/L are of diagnostic support for CF, values between 30 and 59 mmol/L are considered borderline and require further testing, while values below 29 mmol/L are generally regarded as nonindicative of disease.^17,18^ Measurement of sweat chloride concentration remains the gold standard for CF diagnosis and is typically performed using the quantitative pilocarpine iontophoresis test (QPIT), introduced by Gibson and Cooke, which indirectly evaluates CFTR function.^19^ While clinically effective, this method requires specialized equipment, trained personnel, and relatively long analysis times, and may suffer from insufficient sweat volumes, particularly in newborns and infants.^20,21^ Moreover, conventional sweat testing is not well suited for frequent monitoring, therapeutic response assessment, or in vitro functional studies aimed at personalized treatment selection.^22,23^ These limitations have motivated the development of alternative approaches for chloride quantification that are faster, less sample-demanding, and adaptable to experimental models.^24^ In this context, direct measurement of chloride secreted by epithelial cells represents a physiologically relevant strategy to assess CFTR function and pharmacological rescue, especially for theratyping and personalized medicine applications.^25,26^

Laboratory-based chloride detection encompasses diverse analytical approaches including ion chromatography,^27^ spectrophotometry,^28^ fluorescence-based methods achieving micromolar detection,^29^ and ion-selective electrodes with near-Nernstian response.^30^ These conventional methods share common advantages including high sensitivity and selectivity, excellent reproducibility with standardized protocols and accurate quantification. However, these approaches are typically labor-intensive, require specialized instrumentation and trained personnel, and are poorly suited for frequent measurements or decentralized testing.^31^ In the context of CF, sample collection itself represents a major limitation, particularly in newborns and infants, where sweat volumes are often insufficient and skin fragility further complicates analysis.^20,21^ As a result, conventional chloride testing remains largely confined to specialized clinical centers and is not ideal for repeated monitoring or functional assessment of therapeutic response.

These limitations have catalyzed innovation in alternative electrochemical chloride sensors combining microfluidics, flexible electronics, and miniaturized detection platforms.^24,32,33^ In this context, iontophoresis-based strategies have emerged as effective approaches for controlled sweat stimulation at the point-of-care. Recent studies have demonstrated the integration of iontophoresis within wearable and miniaturized platforms, enabling localized sweat induction coupled with real-time analysis of biomarkers. For example, fully integrated systems combining iontophoresis, microfluidics, and electrochemical sensing have been reported for on-body monitoring of metabolites and electrolytes, allowing continuous and noninvasive analysis.^34^ Similarly, wearable platforms incorporating iontophoretic interfaces have enabled programmable sweat extraction and in situ detection of analytes such as glucose and chloride, with direct relevance for cystic fibrosis diagnostics.^35^ In addition, tattoo-based iontophoretic biosensing systems have demonstrated the feasibility of coupling sweat stimulation and electrochemical detection in a fully integrated and wireless format.^36^ Ray et al. (2021) developed a nonsticky sweat sticker patch that directly tracks chloride levels from the skin, producing chloride concentrations equivalent to conventional analysis, with low sample leakage risk and improved infant collection comfort.^37^ In the same manner, la Grasta et al. (2023) developed a miniaturized chloride sensor using an ion-sensitive transistor to directly convert sweat chloride concentrations to electrical signals for real-time monitoring of chloride levels. This minimizes errors in the conventional testing and shows that electrochemical sensing can offer faster, easier, and more accurate chloride analyses, making them attractive for point-of-care (POC) and personalized monitoring applications.^38–40^

In this context, the present study aims to develop and validate a polyester-based screen-printed electrode (SPE) functionalized with silver nanoparticles (AgNPs) for electrochemical chloride detection, with a specific focus on applications relevant to CF theratyping. The platform was applied to quantify chloride in chloride-free buffer placed in contact with the apical surface of differentiated epithelial cell cultures carrying different CFTR pathogenic variants. For each genotype, measurements were performed under untreated (NT) conditions and after treatment with the CFTR modulator combination elexacaftor/tezacaftor/ivacaftor (ETI, commercially known as Trikafta). Chloride concentrations obtained by SPE using a standard addition approach were further processed to calculate standardized chloride secretion (nmol/cm^2^/h), based on the final apical fluid volume. The functional response to ETI was evaluated through absolute values, fold increase (ETI/NT), and correlation analyses, demonstrating that SPE-based chloride quantification provides a rapid, low-cost, and physiologically relevant readout of CFTR activity in patient-derived epithelial models.

## Experimental Section

### Reagents and Equipment

Potassium chloride (KCl) and silver nanoparticles (AgNPs, 10 nm particle size, 0.02 mg/mL) were purchased from Merck Life Science (St. Louis, MO, USA). Milli-Q water was produced in-house to 18 MΩ/cm quality, using a Milli-Q eq 7015 Ultrapure Water Purification System (Darmstadt, Germany). A chloride-free buffer (Cl^–^ -free buffer, pH = 7.0) was prepared in-house with the following composition: 140 mM NaNO_3_, 5 mM KNO_3_, 1 mM Mg­(NO_3_)_2_, 1.5 mM Ca­(NO_3_)_2_, 5 mM HEPES, 5 mM glucose (Sigma-Aldrich (Milan, Italy)) in ultrapure water. Biological samples consisted of Cl^–^ -free buffer placed in contact with the apical side of airway epithelial cells with different CFTR genotypes, including F508del/F508del, L1077P/W1282X, L1077P/L1077P, F508del/S589I, and G85*E*/2183AA > G (Table S1). For each genotype, measurements were performed under untreated (NT) conditions and after treatment with the CFTR modulator combination elexacaftor/tezacaftor/ivacaftor (ETI).

Conductive inks (Ag/AgCl and graphite) were purchased from Sun Chemical (USA). Flexible polyester film (HT5 Autostat) was kindly provided by MacDermid Alpha (UK) and MacDermid Performance Solutions Italiana (Italy). All the electrochemical measurements were carried out with the use of a portable EmStat4S (PalmSens, Netherlands), connected to a laptop. Current responses were recorded and displayed by using the dedicated application PSTrace (5.11) by PalmSens BV.

### Flexible Screen-Printed Electrode Preparation

Electrodes were fabricated in-house on flexible polyester film through the use of a semiautomatic screen-printer (Serilive, Italy). Polyester substrates allow the production of robust, lightweight, and bendable electrochemical strips suitable for decentralized and point-of-care applications. The three-electrode configuration was printed using custom-designed masks, with Ag/AgCl conductive ink employed for the electrical tracks and reference electrode, while carbon ink was used for both the working and counter electrodes. After printing, the strips were thermally cured at 100 °C for 30 min to ensure the polymerization and the electrochemical stability of the inks. The working electrode had a diameter of 0.4 cm, and the overall dimensions of each strip were approximately 2.5 × 1 cm (height × width). To confine the sample and prevent unwanted diffusion toward the connector, an adhesive film was applied to define and seal the working area.^41^

### Sensing Principle and Procedure

The sensing platform is based on polyester-supported SPEs modified with silver nanoparticles (AgNPs). A volume of 2 μL of the AgNP suspension was drop-cast onto the working electrode and allowed to dry at room temperature. The presence of AgNPs is essential for chloride detection: silver reacts with chloride ions to form soluble silver chloride (AgCl) according to the reaction:^42^

$${\mathrm{Ag}}^{0}+{\mathrm{Cl}}^{-}\to \mathrm{AgCl}\left(s\right)+e^{-}$$

This electrochemical conversion produces a characteristic oxidation peak whose current intensity is proportional to the chloride concentration in the sample (Figure 1).^43^

**Figure 1:**
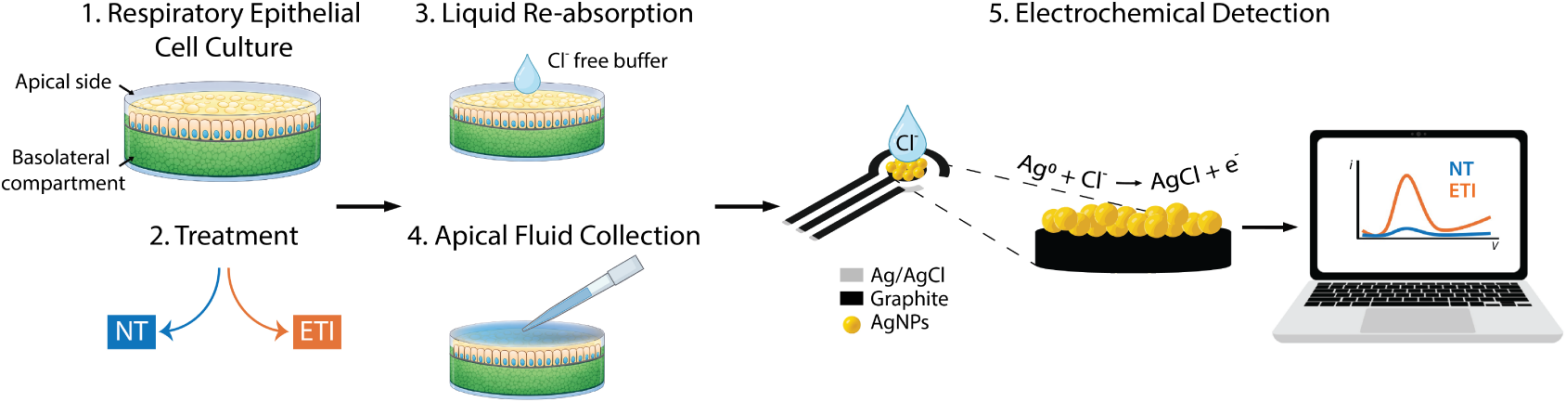
Schematic workflow for electrochemical chloride quantification in cystic fibrosis epithelial cell models: (1) respiratory epithelial cell culture at the air–liquid interface (ALI), (2) treatment with NT or ETI, (3) apical liquid reabsorption using Cl^–^ -free buffer, (4) apical fluid collection, and (5) electrochemical detection of chloride using AgNPs-modified SPEs.

The AgNP-modified SPEs were first evaluated using standard KCl solutions (0–30 mM) prepared in Milli-Q water and, subsequently, in the Cl^–^ -free buffer to mimic the ionic environment of biological samples. For each measurement, 50 μL of solution were deposited onto the working area of the SPE and analyzed by Differential Pulse Voltammetry (DPV). The voltammetric scans were recorded in the range from −0.30 to +0.50 V, with a scan rate of 0.10 V s^–1^. Under these conditions, chloride produced a reproducible oxidation peak centered at +0.22 V, which was used as the analytical signal for quantification.^44^

### Biological Samples from CFTR-Deficient Epithelial Cell Models

Cell cultures and fluid measurement were performed as already described and briefly reported below.^5,45^ The methodology to obtain stem-like long-term patient-specific airway epithelial cells from nasal brushing of pwCF consists in the coculturing of nasal primary epithelial cells with irradiated Swiss 3T3 mouse fibroblasts as feeder layer, in the presence of ROCK inhibitor Y-27632, at 37 °C in 5% CO_2_. After 4 weeks under appropriate culture conditions, they underwent differentiation into mucociliary pseudostratified respiratory epithelium. This is achieved by using the air–liquid interface (ALI) method, seeding cells in transwell porous supports that leaves the apical side of cells exposed to air wherein the basolateral side remains in contact with the medium (Figure 1).

After differentiation in ALI conditions, cells were left untreated or treated with ETI for 48 h in the basolateral chamber. Medium with drugs was replenished in the basolateral chambers and cells were washed three times (apical chamber) with 500 μL of Cl^–^ -free buffer. After washing, the apical side of the epithelium was covered with 200 μL of Cl^–^ -free buffer, overlaid with 300 μL of mineral oil to prevent evaporation. Cultures were maintained to allow liquid reabsorption and ion exchanges in the epithelium for 48 h. Then, the apical fluid was carefully removed, separated through brief centrifugation and the residual volume of aqueous phase was collected and measured in each sample (Figure S1). Cultures were performed in triplicate for each genotype and experimental condition.

### Quantification of Chloride, Data Processing, and Statistical Analysis

For each genotype, three independent biological aliquots obtained from epithelial cell cultures were analyzed. Each aliquot was diluted to 20% (v/v) in Cl^–^ -free buffer and measured in triplicate using three independent SPEs. Chloride concentrations reported in this work represent the mean value obtained from the three biological replicates and the corresponding SPE technical replicates. Chloride concentration in biological samples was determined by differential pulse voltammetry using the standard addition method. The standard addition method was performed in Cl^–^ -free buffer, which served as the calibration matrix to better mimic the ionic environment of the biological samples and ensure consistency between calibration conditions and sample matrix. For each sample, 50 μL of the diluted apical fluid (20% v/v in Cl^–^ -free buffer) were deposited onto the SPE surface, and successive additions of a KCl standard solution were performed to obtain the analytical regression curve directly in matrix.

To enable comparison across samples with different epithelial genotypes, a standardized chloride secretion calculation was carried out, expressed in nmol/cm^2^/h, based on the SPE-derived chloride concentrations. The calculation accounted for: (i) the final apical surface liquid volume, (ii) the total amount of chloride present in the sample, (iii) the culture surface area, and (iv) the experiment duration. Standardized chloride secretion (nmol/cm^2^/h) was calculated according to the following equation:

Chloridesecretion=((C×V)/A)/t

where C is the chloride concentration expressed in nmol/μL (obtained by converting the electrochemically measured concentration from mM), V is the final apical fluid volume (μL), A is the culture surface area (cm^2^), and t is the incubation time (h).

Fold-changes between treated (ETI) and untreated (NT) conditions were computed for both raw concentration data and standardized secretion values. Correlation analyses between chloride concentration and chloride secretion were performed using Pearson’s correlation coefficient (r), its coefficient of determination (r^2^), and associated *p*-values. Statistical analyses were performed using paired Student’s *t* tests to evaluate differences between NT and ETI conditions. Differences were considered statistically significant at *p* < 0.05 (*p* < 0.05 = *, *p* < 0.01 = **, *p* < 0.001 = ***, *ns* = not significant).

## Results and Discussion

### Optimization of the AgNPs-Modified SPE and Analytical Performance toward Chloride Detection

The sensing performance of the polyester-based SPE was first optimized by evaluating the effect of the amount of AgNPs drop-cast onto the working electrode surface. The use of AgNPs plays a key role in enhancing the electrochemical response toward chloride detection. Compared to bulk silver, AgNPs provide a higher surface-to-volume ratio, increasing the number of reactive sites and improving electron-transfer kinetics.^42^ This results in an enhanced analytical signal and improved sensitivity toward chloride ions. As shown in Figure S2, the DPV current response recorded in the presence of 10 mM KCl, increased markedly after modification with AgNPs, confirming their key role in promoting the electrochemical recognition of Cl^–^ through the formation of AgCl at the electrode surface.^44^ In particular, the deposition of 2 μL of AgNPs resulted in a significant enhancement of the analytical signal compared to the bare electrode, while no statistically significant difference was observed between 2 and 4 μL. Conversely, a further increase of the AgNPs volume to 8 μL led to a decrease in current intensity, likely due to partial blocking of the electrode surface and hindered mass transport. Based on these results, 2 μL of AgNPs was selected as the optimal modification volume and used for all subsequent experiments. Following optimization, the analytical response of the AgNPs-modified SPE toward chloride was evaluated by recording DPV measurements at increasing concentrations of KCl. Calibration experiments were first performed in aqueous solutions over the 0–30 mM concentration range. As shown in Figure 2A, the chloride oxidation peak, centered at approximately +0.22 V vs Ag/AgCl, increased proportionally with chloride concentration, yielding a linear response described by the equation y = 0.25x + 0.17 (R^2^ = 0.99). The limit of detection (LOD) and the limit of quantification (LOQ) were calculated as 3σb/slope and 10σb/slope, where σb is the standard deviation of the blank measurements, and resulted equal to 0.07 mM and 0.2 mM, respectively. This behavior confirms the suitability of the AgNPs-modified SPE for quantitative chloride detection under controlled conditions.

**Figure 2:**
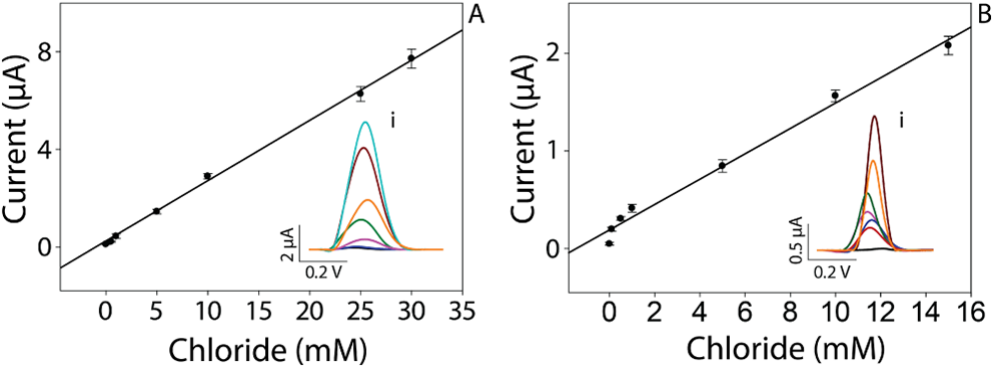
(A) Calibration curve of the AgNPs-modified SPE recorded at increasing KCl concentrations (0–30 mM) prepared in water. (B) Calibration curve of the AgNPs-modified SPE recorded at increasing KCl concentrations (0–15 mM) in Cl^–^ -free buffer. Data are reported as mean ± standard deviation (*n* = 4). The insets (i) show the corresponding DPV current peaks for chloride oxidation measured at approximately +0.22 V vs Ag/AgCl.

To better mimic the experimental conditions used for biological measurements, the same calibration protocol was then applied in a Cl^–^ -free buffer. As reported in Figure 2B, a linear increase of the DPV peak current was again observed with increasing KCl concentration (0–15 mM), demonstrating that the sensing platform maintains its analytical performance also in a more complex ionic matrix. The linearity was described by the following equation y = 0.13x + 0.19 (R^2^ = 0.98), with a reached LOD of 0.08 mM and a LOQ of 0.3 mM, calculated as before. Good repeatability was achieved, with relative standard deviation (RSD) values of 7% (n = 6) and well-defined peak shapes, for both the conditions tested.

As expected, higher absolute current responses were observed in water compared to the buffer, likely due to matrix effects and the presence of competing ions in the latter. As reflected also by the slope of the calibration curves, the sensitivity decreased in Cl^–^ -free buffer compared to water, consistently with increased ionic strength and matrix effects. Nevertheless, in both media the sensor exhibited a linear response over the investigated concentration range, demonstrating its suitability for the quantitative determination of chloride in complex biological matrices, which was subsequently performed using the standard addition method.

### Quantification of Chloride in CFTR-Deficient Epithelial Cell Cultures

The developed AgNPs-modified SPE was applied to the quantification of chloride in biological samples consisting of Cl^–^ -free buffer collected from the apical surface of CFTR-deficient epithelial cells. Samples derived from different CFTR mutated genotypes were analyzed under untreated (NT) conditions and after treatment with the CFTR modulator combination elexacaftor/tezacaftor/ivacaftor (ETI). Due to the limited volume of the collected apical surface liquid, all samples were diluted to 20% (v/v) in Cl^–^ -free buffer prior to electrochemical analysis. Chloride quantification was performed using the standard addition method, which allows accurate determination of analyte concentration in complex matrices while minimizing matrix-related effects.

For each diluted sample (NT and ETI), three successive additions of KCl (0.1, 0.5, and 1.0 mM) were carried out. This concentration range was selected to fall within both the linear response range of the sensor and relevant chloride levels. The use of standard addition was also necessary to enable measurements in triplicates SPE replicates, given the limited amount of biological material available. For each genotype and condition the values reported represent the average of three independent biological samples (a, b, c), each analyzed using three SPE replicates. Standard addition plots for all analyzed samples showed a linear increase of the chloride oxidation peak current as a function of the added chloride concentration, with good linearity in all cases (R^2^ > 0.97), confirming the suitability of the proposed approach for chloride quantification in complex biological matrices. All standard addition plots are reported in the Supporting Information (Figures S3–S9). The quantified chloride concentrations obtained for NT and ETI-treated samples are shown in Figure 3A, with corresponding values reported in Table S2 (SI).

**Figure 3:**
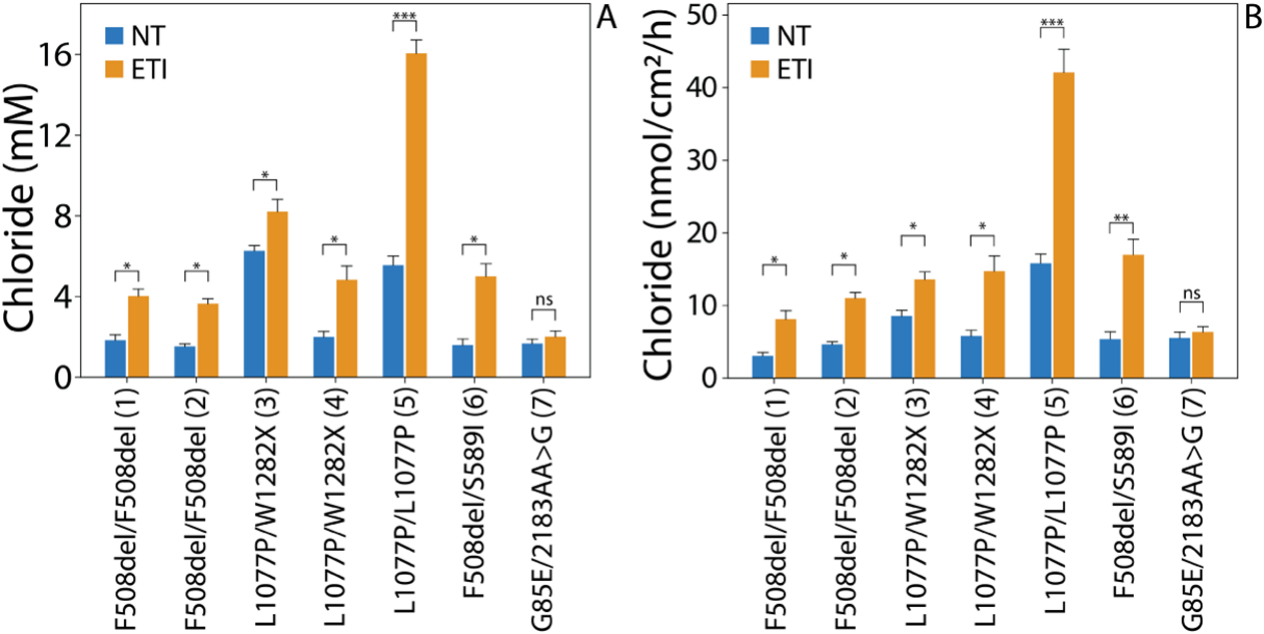
Quantification of chloride in apical fluid collected from CFTR-deficient epithelial cell cultures under untreated (NT) conditions and after treatment with elexacaftor/tezacaftor/ivacaftor (ETI). (A) Chloride concentration (mM) determined by differential pulse voltammetry using the standard addition method on samples diluted at 20% in Cl^–^ -free buffer. (B) Standardized chloride secretion expressed as nmol/cm^2^/h, calculated by normalizing the measured chloride concentrations to the final apical surface liquid volume, culture surface area, and experimental time. Data are reported as mean ± SD, calculated from three independent biological aliquots per genotype and three SPE replicates per aliquot. Statistical significance between NT and ETI conditions was assessed separately for each genotype using a paired Student’s *t* test (*p* < 0.05 = *, *p* < 0.01 = **, *p* < 0.001 = ***, *ns* = not significant).

### Normalization of Chloride Secretion and Correlation Analysis

To further strengthen the physiological relevance of the electrochemical measurements, the chloride concentrations quantified by SPE were subsequently normalized to express chloride secretion as nmol per unit area of cell culture and time of experiment (nmol/cm^2^/h). This normalization was performed by multiplying the measured chloride concentration by the final volume of the apical surface liquid collected at the end of the experiment, thus obtaining the total amount of secreted chloride (nmol), which was then divided by the surface area of the culture well and by the duration of the experiment. This standardized parameter was introduced to account for the coupling between CFTR-mediated chloride secretion and epithelial fluid movement. Indeed, an increase in CFTR activity is typically accompanied by enhanced water secretion, which may partially mask functional recovery when chloride is evaluated solely in terms of concentration.

As shown in the comparative analyses (Figure 3A,B), both untreated (NT) and ETI-treated samples displayed consistent trends when chloride transport was expressed either as concentration or as standardized secretion rate, indicating good agreement between the two readouts. The statistical significance of the comparison between NT and ETI conditions was confirmed in both raw and normalized data, with significance levels ranging from *p < 0.05 to ***p < 0.001, while no significant change was observed for G85*E*/2183AA > G. Importantly, the magnitude of chloride increase following ETI treatment was genotype-dependent, reflecting differences in intrinsic CFTR function and modulator responsiveness. Samples harboring pathogenic variants known to be highly responsive to ETI, such as F508del/F508del and L1077P/L1077P, exhibited a marked enhancement of chloride secretion after treatment.^16,46^ In contrast, genotypes containing at least one allele with limited CFTR rescue potential (e.g., L1077P/W1282X and G85*E*/2183AA > G) showed smaller improvements. These observations are in line with the known variability of CFTR modulator effects across different pathogenic variants, where certain variants permit substantial functional rescue upon modulator treatment while others do not.^47,48^ This genotype-specific behavior underscores the value of functional assays in personalized evaluation of CFTR modulator efficacy (theratyping).

Importantly, the fold increase (ETI/NT ratio) calculated from direct chloride concentration measurements closely mirrored that obtained from the standardized secretion values expressed as nmol/cm^2^/h (Figure 4A). This agreement indicates that, across the different genotypes analyzed, the relative response to ETI treatment is consistently captured by both approaches. As before, the largest fold increases were observed in genotypes known to be highly responsive to CFTR modulators, such as F508del/F508del and L1077P/L1077P, while genotypes carrying pathogenic variants associated with limited CFTR rescue, including G85*E*/2183AA > G, displayed minimal changes following treatment. These findings further support the biological relevance of the electrochemical readout.

**Figure 4:**
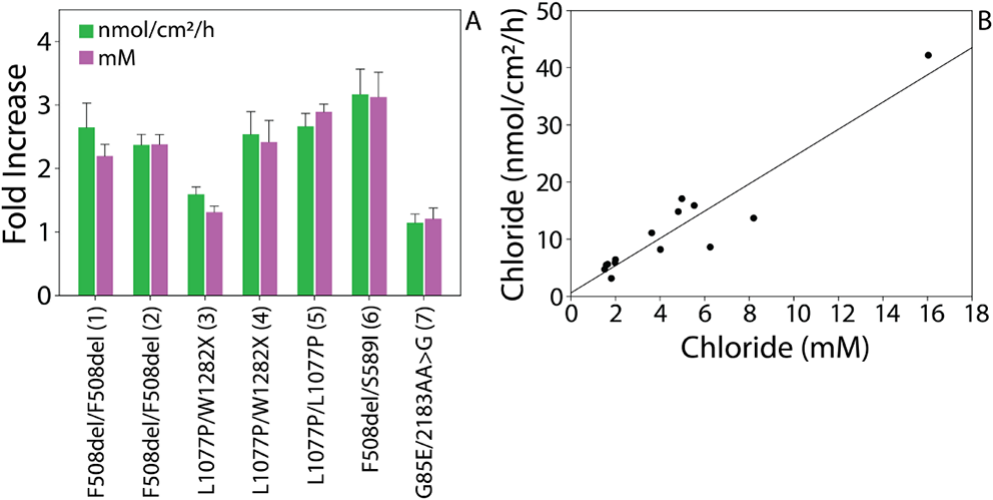
(A) Fold increase (ETI/NT ratio) of chloride levels calculated from direct electrochemical measurements (mM) and from standardized chloride secretion values (nmol/cm^2^/h) for the different CFTR genotypes analyzed. Data are reported as mean ± SD. (B) Correlation between chloride concentration measured by SPE (mM) and standardized chloride secretion (nmol/cm^2^/h) across all analyzed samples. The strong linear relationship (R^2^ = 0.98) demonstrates that direct electrochemical chloride quantification reliably reflects normalized secretion rates.

Correlation analysis between chloride concentration measured by SPE and the corresponding standardized chloride secretion (nmol/cm^2^/h) revealed a very strong positive linear relationship (Figure 4B). Linear regression yielded the equation y = 2.6x + 0.8 with an excellent coefficient of determination (R^2^ = 0.98), indicating that nearly all the variability in normalized chloride secretion is explained by the directly measured chloride concentration. Pearson’s correlation analysis confirmed this result, showing a near-perfect correlation (r = 0.99, *p* < 0.001), thus demonstrating the high statistical significance of the association.

These results indicate that, despite the theoretical influence of fluid movement on chloride concentration, direct electrochemical quantification by AgNPs-modified SPE reliably reflects normalized chloride secretion across the investigated genotypes and treatment conditions. The strong statistical agreement between the two metrics supports the use of concentration-based measurements as a valid and robust method for CFTR-dependent chloride transport, enabling reliable discrimination between ETI-responsive and nonresponsive genotypes.

It is important to note that, in the context of epithelial cell culture models, straightforward alternative methods for chloride quantification are limited, particularly when dealing with small apical volumes. In this regard, the proposed electrochemical approach represents a practical and effective solution for chloride analysis in low-volume biological samples.

This versatility, combined with the simplicity, low cost, and rapidity of the approach, highlights the potential utility of the proposed platform for CFTR functional assessment and theratyping applications.

## Conclusions

In this work, a nanomodified electrochemical sensor was implemented to improve the theratyping in CF models. Chloride levels were quantified in the apical surface of differentiated CFTR-deficient respiratory epithelial cell cultures derived from pwCF with different CFTR genotypes. The quantified chloride levels were used to calculate standardized chloride secretion rates (nmol/cm^2^/h), providing a physiologically meaningful metric of CFTR function. Treatment with the CFTR modulator combination ETI, namely elexacaftor, tezacaftor and ivacaftor, induced genotype-dependent increases in chloride secretion, clearly distinguishing responsive from less responsive genotypes. Importantly, a strong correlation between directly measured chloride concentration and normalized secretion rates was observed, indicating that direct electrochemical measurements are sufficient to capture treatment-induced functional changes without requiring complex postanalytical corrections. Overall, this study demonstrates that the proposed SPE-based platform represents a rapid, low-cost, and reliable analytical tool for functional assessment of CFTR activity in patient-derived respiratory epithelial models. The approach is well suited for theratyping applications and could support preclinical evaluation of CFTR modulator efficacy, offering a practical bridge between analytical chemistry and personalized medicine in CF research.

## Supplementary Material





## Notes

Table S1: reports CFTR-deficient epithelial cell samples with their genotypes and treatment conditions; Table S2: provides chloride concentrations measured by AgNPs-modified SPE in biological samples from CFTR-deficient epithelial cell models under untreated (NT) conditions and after ETI treatment; Figures S1–S9: display images of the employed cell cultures, electrochemical characterization of AgNP-modified electrodes, and standard addition plots for chloride quantification in apical fluid samples from different CFTR genotypes under both untreated and ETI-treated conditions (PDF)
The AVI file reports representative frames showing differentiated cells under ALI conditions with beating cilia (AVI)
