# Supplementary material for: Toward Precision Electrochemical Sensing of CFTR Function in Cystic Fibrosis Models
Source: Anal Chem. 2026 Jun 4;98(23):16805–12. doi: 10.1021/acs.analchem.6c00392 (PMC13276835; doi:10.1021/acs.analchem.6c00392)
Supplement: Supplementary file 1 [file ac6c00392_si_001.pdf]

Supporting information

Toward precision electrochemical sensing of CFTR function in cystic fibrosis models

Antonella Miglione[1‡*], *Giovanna Blaconà*[2‡], *Sima Singh*[3], *Stefania Lo Cicero*[4], *Andrea Fuso*[2,5], *Giuseppe Cimino*[6], *Adriana Eramo*[4], *Umberto Malapelle*[1], *Marco Lucarelli*[2,7§*], *Stefano Cinti*[3,8,9§*]

[1]Department of Public Health, University Federico II of Naples, Naples, Italy.

[2]Department of Experimental Medicine, Sapienza University of Rome, Rome, Italy.

[3]Department of Pharmacy, University of Naples Federico II, Via D. Montesano 49, Naples, 80131, Italy.

[4] Department of Oncology and Molecular Medicine, Istituto Superiore di Sanità, 00161 Rome, Italy.

[5]CRiN, Center for Research in Neurobiology, Sapienza University of Rome, Rome, Italy.

[6]Cystic Fibrosis Centre, AOU Policlinico Umberto 1, 00155 Rome, Italy.

[7]Pasteur Institute Cenci Bolognetti Foundation, Sapienza University of Rome, Rome, Italy.

[8]Bioelectronics Task Force at University of Naples Federico II, Via Cinthia 21, Naples, 80126, Italy.

[9]Sbarro Institute for Cancer Research and Molecular Medicine, Center for Biotechnology, College of Science and Technology, Temple University, Philadelphia, PA, 19122, USA.

[‡] *These authors are co–first authors.*

[§] *These authors are co–last authors.*

*These authors are co-corresponding authors

E-mail: stefano.cinti@unina.it; marco.lucarelli@uniroma1.it; antonella.miglione@unina.it

Table S1. CFTR-deficient epithelial cell samples analyzed, reporting sample ID, genotype, and treatment condition (NT or ETI - Trikafta® treatment).

Sample ID	CFTR genotype	Treatment condition
1	F508del/F508del	NT
		ETI
2	F508del/F508del	NT
		ETI
3	L1077P/W1282X	NT
		ETI
4	L1077P/W1282X	NT
		ETI
5	L1077P/L1077P	NT
		ETI
6	F508del/S589I	NT
		ETI
7	G85E/2183AA>G	NT
		ETI

Table S2. Chloride concentration measured by AgNPs-modified SPE in biological samples from CFTR-deficient epithelial cell models under untreated (NT) conditions and after ETI treatment. Data are reported as mean on 3 biological samples ± SD (n = 3).

CFTR genotypes	Condition	Chloride (mM) ± SD
F508del/F508del (1)	NT	1.8 ± 0.3
	ETI	4.0 ± 0.3
F508del/F508del (2)	NT	1.5 ± 0.1
	ETI	3.7 ± 0.2
L1077P/W1282X (3)	NT	6.3 ± 0.3
	ETI	8.2 ± 0.6
L1077P/W1282X (4)	NT	2.0 ± 0.3
	ETI	4.8 ± 0.7
L1077P/L1077P (5)	NT	5.6 ± 0.5
	ETI	16.1 ± 0.7
F508del/S589I (6)	NT	1.6 ± 0.3
	ETI	5.0 ± 0.6
G85E/2183AA>G (7)	NT	1.7 ± 0.2

	ETI	2.0 ± 0.3

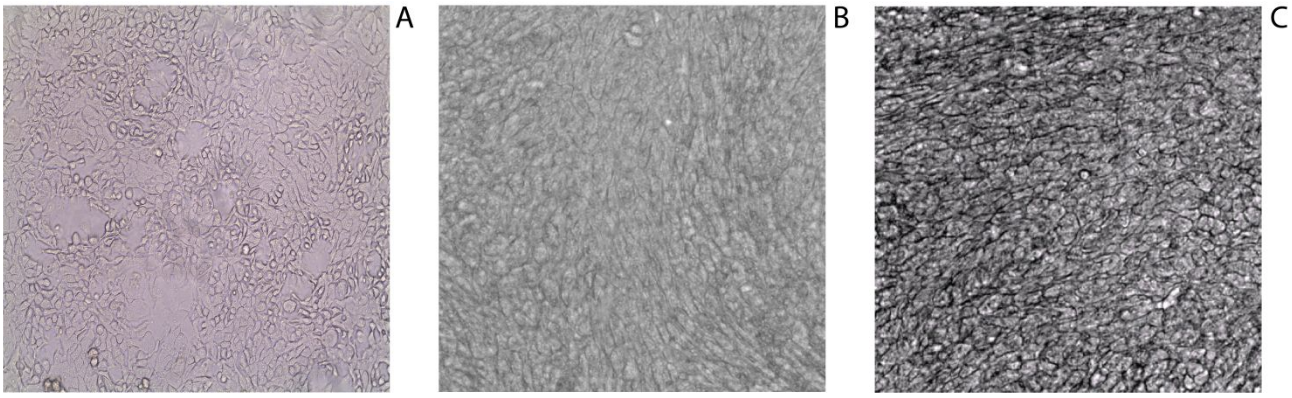

Figure S1. Cell cultures of airway epithelial cells from nasal brushing, under the optical microscope at 20X magnification. A) Picture of undifferentiated cells under CRC culture conditions; B) Picture of differentiated cells under ALI culture conditions; C) Representative frame from a video showing differentiated cells under ALI conditions with beating cilia (see Movie S1).

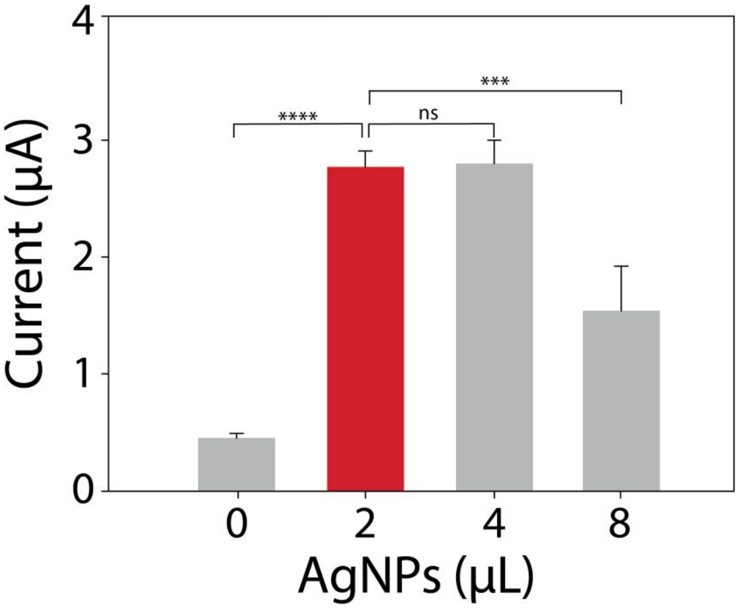

Figure S2. Differential pulse voltammetry (DPV) current response in 10 mM KCl for electrodes modified with increasing volumes of AgNPs (0, 2, 4, and 8 µL). Statistical significance is indicated as $p < 0.001$ =***; $p < 0.0001$ =****; ns: not significant).

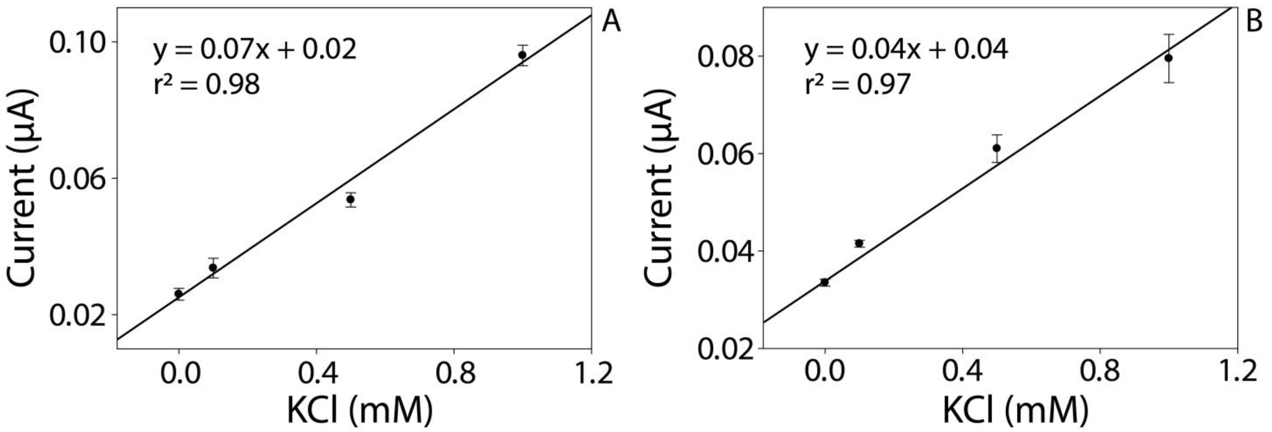

Figure S3. Standard addition plots obtained by DPV for chloride quantification in apical fluid samples from the F508del/F508del (1) genotype. A) Untreated (NT) sample and (B) ETI-treated sample. Increasing concentrations of KCl (0.1–1.0 mM) were added directly to the diluted sample (20% v/v in Cl^--free buffer). Each data point represents the mean ± SD of three independent SPE measurements. Linear regression equations and corresponding r^2 values are reported in each panel.

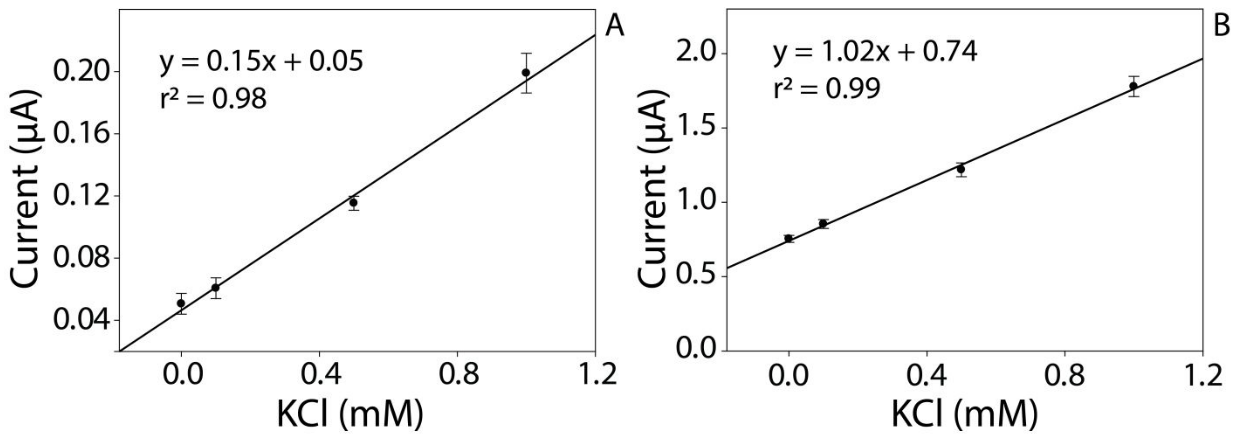

Figure S4. Standard addition plots obtained by DPV for chloride quantification in apical fluid samples from the F508del/F508del (2) genotype. A) Untreated (NT) sample and (B) ETI-treated sample. Increasing concentrations of KCl (0.1–1.0 mM) were added directly to the diluted sample (20% v/v in Cl^--free buffer). Each data point represents the mean ± SD of three independent SPE measurements. Linear regression equations and corresponding r^2 values are reported in each panel.

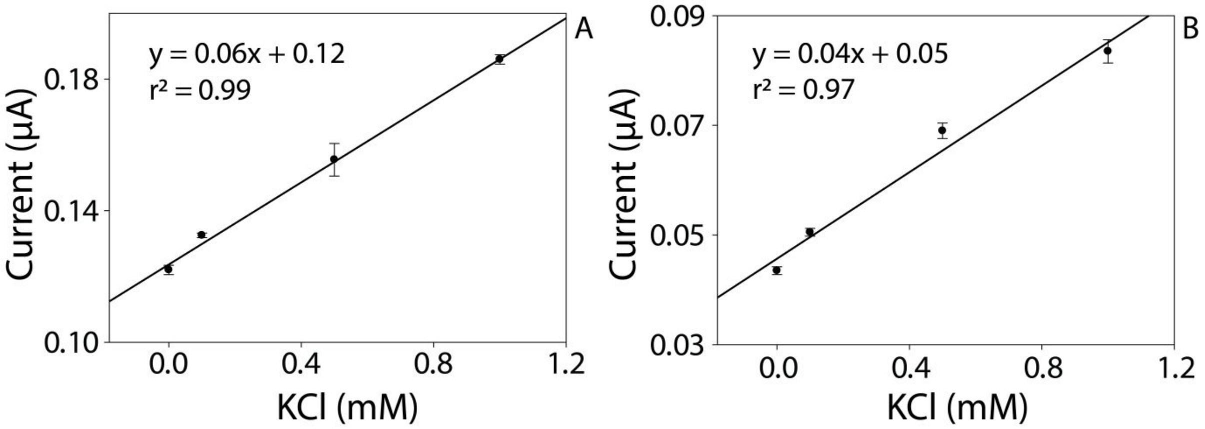

Figure S5. Standard addition plots obtained by DPV for chloride quantification in apical fluid samples from the L1077P/W1282X (3) genotype. A) Untreated (NT) sample and (B) ETI-treated sample. Increasing concentrations of KCl (0.1–1.0 mM) were added directly to the diluted sample (20% v/v in Cl^--free buffer). Each data point represents the mean ± SD of three independent SPE measurements. Linear regression equations and corresponding r^2 values are reported in each panel.

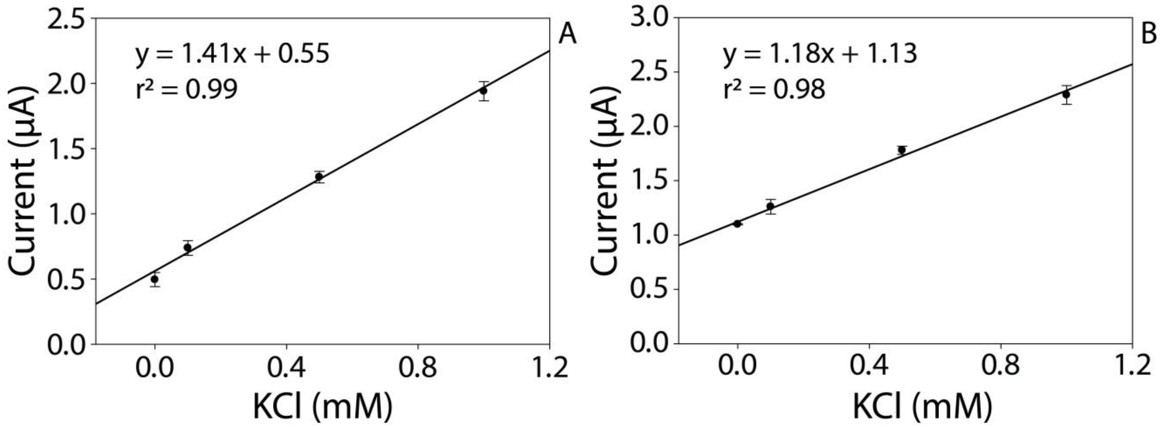

Figure S6. Standard addition plots obtained by DPV for chloride quantification in apical fluid samples from the L1077P/W1282X (4) genotype. A) Untreated (NT) sample and (B) ETI-treated sample. Increasing concentrations of KCl (0.1–1.0 mM) were added directly to the diluted sample (20% v/v in Cl^--free buffer). Each data point represents the mean ± SD of three independent SPE measurements. Linear regression equations and corresponding r^2 values are reported in each panel.

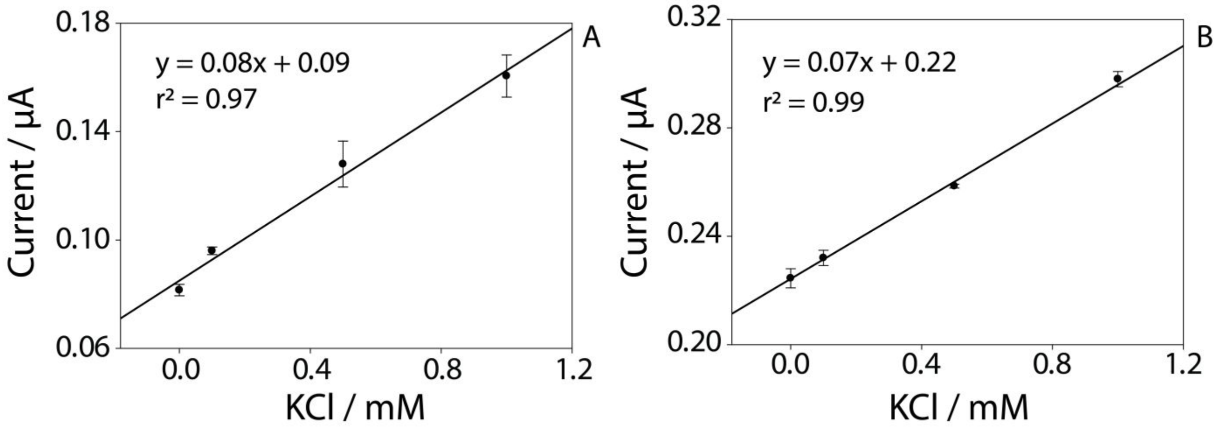

Figure S7. Standard addition plots obtained by DPV for chloride quantification in apical fluid samples from the L1077P/L1077P (5) genotype. A) Untreated (NT) sample and (B) ETI-treated sample. Increasing concentrations of KCl (0.1–1.0 mM) were added directly to the diluted sample (20% v/v in Cl^--free buffer). Each data point represents the mean ± SD of three independent SPE measurements. Linear regression equations and corresponding r^2 values are reported in each panel.

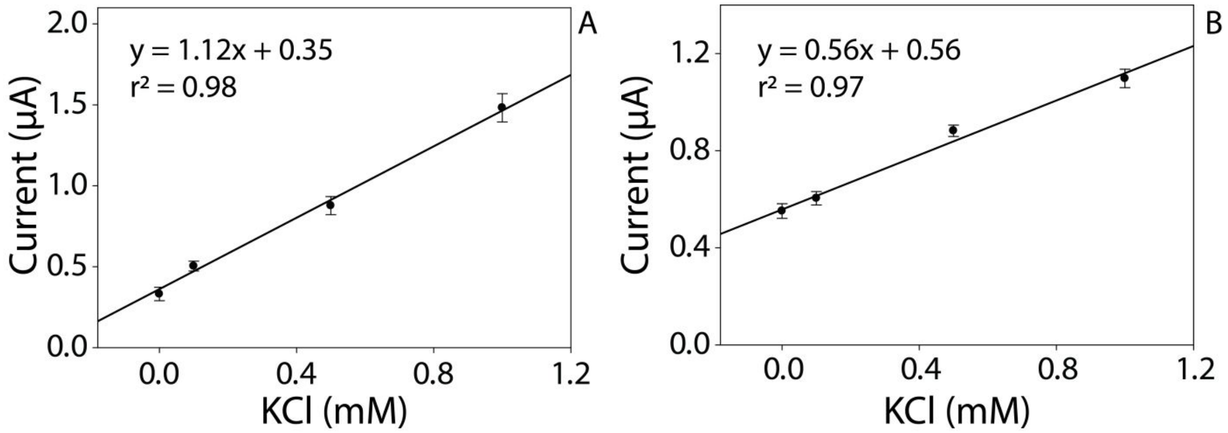

Figure S8. Standard addition plots obtained by DPV for chloride quantification in apical fluid samples from the F508del/S589I (6) genotype. A) Untreated (NT) sample and (B) ETI-treated sample. Increasing concentrations of KCl (0.1–1.0 mM) were added directly to the diluted sample (20% v/v in Cl^--free buffer). Each data point represents the mean ± SD of three independent SPE measurements. Linear regression equations and corresponding r^2 values are reported in each panel.

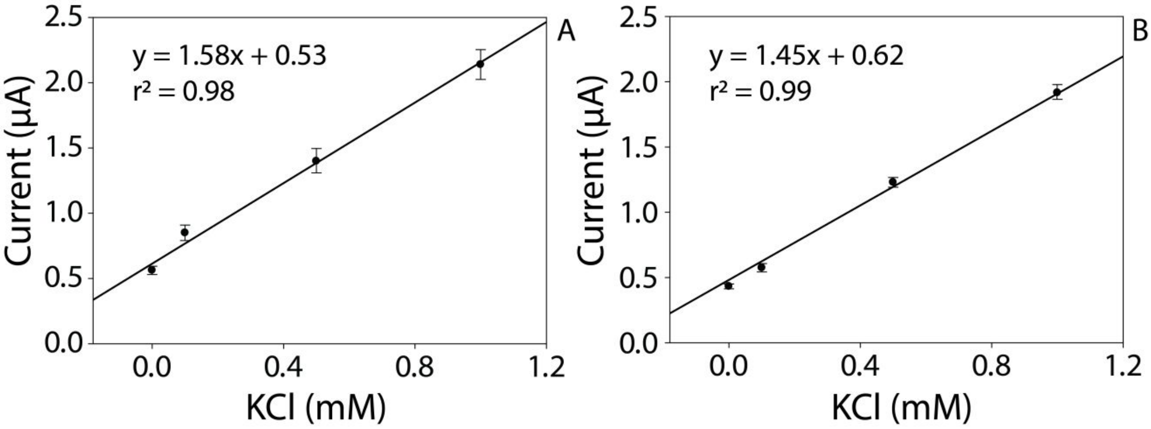

Figure S9. Standard addition plots obtained by DPV for chloride quantification in apical fluid samples from the G85E/2183AA>G (7) genotype. A) Untreated (NT) sample and (B) ETI-treated sample. Increasing concentrations of KCl (0.1–1.0 mM) were added directly to the diluted sample (20% v/v in Cl^--free buffer). Each data point represents the mean ± SD of three independent SPE measurements. Linear regression equations and corresponding r^2 values are reported in each panel.
